# Probiotics and Amelioration of Rheumatoid Arthritis: Significant Roles of *Lactobacillus casei* and *Lactobacillus acidophilus*

**DOI:** 10.3390/microorganisms9051070

**Published:** 2021-05-16

**Authors:** Alok K. Paul, Anita Paul, Rownak Jahan, Khoshnur Jannat, Tohmina A. Bondhon, Anamul Hasan, Veeranoot Nissapatorn, Maria L. Pereira, Polrat Wilairatana, Mohammed Rahmatullah

**Affiliations:** 1 Department of Biotechnology & Genetic Engineering, University of Development Alternative, Dhaka 1207, Bangladesh; alokkpaul@gmail.com (A.K.P.); rownak86@hotmail.com (R.J.); jannat.koli.22@gmail.com (K.J.); afrozebondhon@gmail.com (T.A.B.); anamulhasanoris@gmail.com (A.H.); 2 Department of Pharmacy, University of Development Alternative, Dhaka 1207, Bangladesh; anita.paul1988@gmail.com; 3 School of Allied Health Sciences, World Union for Herbal Drug Discovery (WUHeDD), and Research Excellence Center for Innovation and Health Products (RECIHP), Walailak University, Nakhon Si Thammarat 80160, Thailand; nissapat@gmail.com; 4 CICECO-Aveiro Institute of Materials & Department of Medical Sciences, University of Aveiro, 3810-193 Aveiro, Portugal; mlourdespereira@ua.pt; 5 Department of Clinical Tropical Medicine, Faculty of Tropical Medicine, Mahidol University, Bangkok 73170, Thailand

**Keywords:** rheumatoid arthritis, probiotics, *Lactobacillus*, *Prevotella*, gut microbiota

## Abstract

Rheumatoid arthritis is a chronic autoimmune disorder that can lead to disability conditions with swollen joints, pain, stiffness, cartilage degradation, and osteoporosis. Genetic, epigenetic, sex-specific factors, smoking, air pollution, food, oral hygiene, periodontitis, *Prevotella*, and imbalance in the gastrointestinal microbiota are possible sources of the initiation or progression of rheumatoid arthritis, although the detailed mechanisms still need to be elucidated. Probiotics containing *Lactobacillus spp.* are commonly used as alleviating agents or food supplements to manage diarrhea, dysentery, develop immunity, and maintain general health. The mechanism of action of *Lactobacillus spp.* against rheumatoid arthritis is still not clearly known to date. In this narrative review, we recapitulate the findings of recent studies to understand the overall pathogenesis of rheumatoid arthritis and the roles of *probiotics,* particularly *L. casei* or *L. acidophilus,* in the management of rheumatoid arthritis in clinical and preclinical studies.

## 1. Introduction

Rheumatoid arthritis (RA) is an autoimmune disorder characterized by swollen joints with chronic pain, stiffness, reduced functionality, osteoporosis, and cartilage degradation leading to a situation of disability [1,2]. RA is prevalent in 0.3–1.0% of adults aged 20–40 years globally, and it is very common among older adults aged 75 years or above [3,4]. Women are relatively more susceptible to RA [4]. RA patients experience age-related comorbidities such as diabetes, cardiovascular disorders, nephritis, chronic obstructive pulmonary disease (COPD), psychological distress, osteoporosis, asthma, and cancer, leading to a higher mortality rate [5].

RA is currently treated with nonsteroidal anti-inflammatory drugs (NSAIDs), as these drugs prevent prostaglandin synthesis by inhibiting the cyclooxygenase enzymes (COX-1 and COX-2) and thereby act against inflammation and pain. Drugs in this group include paracetamol, ibuprofen, naproxen, diclofenac, indomethacin, ketoprofen, meloxicam, and many more [6]. Besides NSAIDs, disease-modifying anti-rheumatic drugs like methotrexate are used as a first-line drug for RA treatment, but 20 to 30% of RA patients could not continue its treatment for >12 months because of adverse reactions. The most common adverse effects of the drug are gastrointestinal toxicity (20–70% of RA patients experience nausea, vomiting, diarrhea, mucocutaneous ulcers), hepatotoxicity (~70% of RA patients), pulmonary toxicity, nephrotoxicity, and blood-related toxicity. The drug is teratogenic, carcinogenic, and anemic and causes patient non-compliance and discontinuation of the treatment [7]. Methotrexate treatment can increase the formation of rheumatoid nodules (lumps under the skin) [8]. Biological disease-modifying anti-rheumatic drugs are also widely used to treat RA, but Janus kinase (JAK) inhibitors like baricitinib and upadacitinib may cause venous thromboembolism [9].

Hydroxychloroquine and chloroquine are antimalarial drugs and are also used for the treatment of RA. The drugs prevent lysosomal degradation of antigens (prevents phagocytosis and autophagy pathways), inhibit the immune response by blocking the Toll-like receptor (TLR)-7 and TLR-9 receptors of dendritic cells by blocking the expression of major histocompatibility complex (MHC) class II molecules and thus modulating T-cells- and B-cells-mediated secretions of proinflammatory cytokines (IL-1, IL-6, and TNF-α) [10]. The drugs can produce retinopathy [10,11,12,13]. As the drugs are weak bases and have a long shelf life in the blood (~50 days), co-treatment with certain drugs with narrow therapeutic indices can create systemic toxicity in patients with hepatic and renal impairments [10].

TNF-α, a proinflammatory cytokine produced inside the body mainly by macrophages and lymphocytes, and induces inflammatory responses by activating nuclear factor-B, proteases, and protein kinases [14,15]. The combination of TNF-α inhibitors and methotrexate can reduce the chance of discontinuation of the therapy in patients with RA [16,17]. TNF-α inhibitor can cause pneumonia and bacterial, viral, and fungal infections, mainly in the respiratory tract, urinary tract, gastrointestinal tract, and skin [18,19]. These drugs need subcutaneous or intravenous administration and may create infections at the injection site [14,20]. TNF-α inhibitors may pose a risk for cancer development, although the findings are inconclusive [21,22,23].

Corticosteroids (especially glucocorticoids such as prednisone, hydrocortisone, and dexamethasone) are immunosuppressants and anti-inflammatory drugs, and these are used in combination with the disease-modifying anti-rheumatic drugs. The combined treatment provides a better onset of symptomatic treatment and reduces adverse reactions associated with the treatment of the disease-modifying anti-rheumatic drugs [24,25,26]. Glucocorticoid receptor usually remains in the cytoplasm in an inactive form, but glucocorticoids activate it. The interaction of glucocorticoids and glucocorticoid receptors suppresses the activity of NF-κB and activator protein-1. This interaction, in turn, prevents the expression of inflammatory genes and transcriptions of proinflammatory cytokines (e.g., IL-1β, IL-6, and TNF-α) [24,27]. Glucocorticoids can induce apoptosis of T-cells, neutrophils, and some other activated white blood cells, as well as increase the phagocytic capability of macrophages [27,28]. NF-κB is expressed in osteoclast precursors, and it is a key regulator of osteoclastogenesis [29]. As glucocorticoids suppress NF-κB expression, it may prevent osteoporosis, which is also a comorbidity of RA. Glucocorticoid use over a long time can cause adverse effects such as hyperglycemia, diabetes, weight gain, fat deposition on the upper body parts, and reduced calcium resorption (and osteoporosis), myopathy, and adrenal insufficiency [30]. Briefly, all the drugs available in the clinic RA treatment produce adverse reactions. A detailed understanding of RA pathogenesis will help us to develop alternative management strategies like using food supplements such as probiotics against the disease manifestation of RA.

## 2. Rheumatoid Arthritis: An Inflammatory Pathway

**Figure 1:**
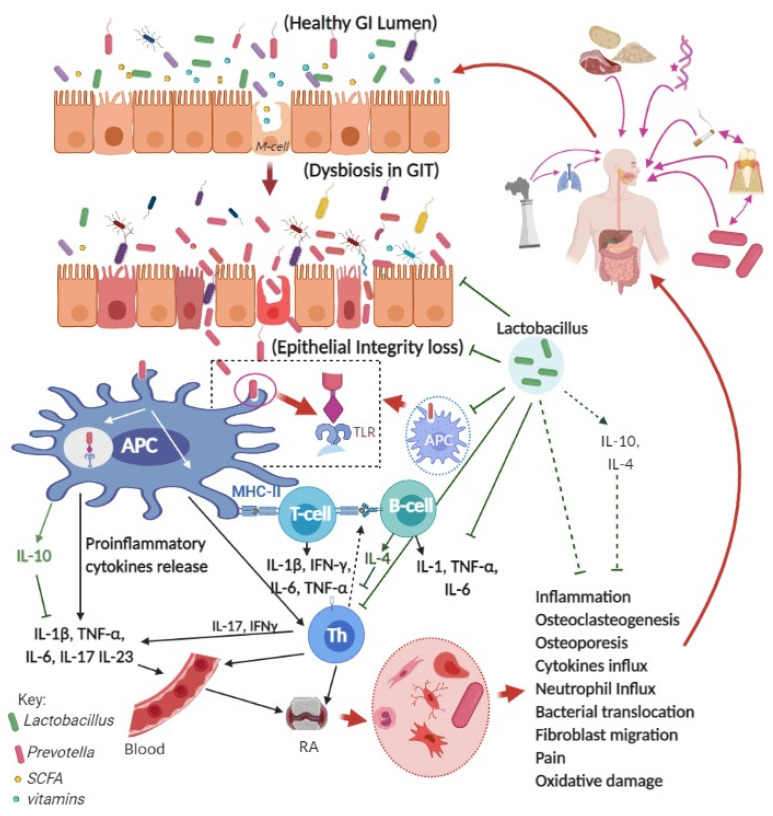
Possible mechanisms of Rheumatoid Arthritis (RA) and the role of *Lactobacillus spp.* Keys: APC, antigen-presenting cell; GI, gastrointestinal, GIT, gastrointestinal tract; IL, Interleukin; TNF-α, tumor necrosis factor alpha; IFN-γ, gamma interferon; M-cell, microfold cell; Th, Helper T cell; T-cell, T-cell lymphocytes, B-cell, B-cell lymphocytes; green rod-shaped bacteria, *Lactobacillus* spp.; red rod-shaped bacteria, *Prevotella* spp.; SCFA, short-chain fatty acids. Figure was made with www.biorender.com (access date: 14 April 2021).

RA manifests with increased fluid retention, neuronal and inflammatory cell migration to the joints with increased secretion of proinflammatory cytokines (IL-1, IL-8, IL-12, IL-15, IL-17, IL-18, IL-29, and TNF-α) [31]. Studies have shown that TNF-α and IL-6 inhibitors are effective against RA [32,33,34,35]. The inflammatory process also results from reduced production of immunomodulatory cytokines like IL-11, IL-13, and IL-10 [31]. Increased ratio of proinflammatory and anti-inflammatory cytokines causes chronic inflammatory conditions like RA, and the process is induced by helper T cell type 1 (Th1) [36,37,38]. Helper T17 (Th17) cells (that defend against extracellular microbes) produce inflammatory response TNF-α, IL-17A, IL-17F, and IFNγ (γ-interferon) that leads to the pathogenesis of RA [39] (Figure 1). RA is influenced by some receptors that can recognize pathogen-associated molecular patterns, such as Toll-like receptors (TLRs), which activate host-defense mechanisms and maintain innate immune responses [40,41,42]. TLRs (especially TLR-2, TLR-3, TLR-4, TLR-5, and TLR-7 in RA) regulate the nuclear factor kappa-B ligand (NF-κB), generation of osteoclasts, and induce increased production of TNF-α, IL-6, IL-12, IL-18, and many other proinflammatory cytokines [40,43]. TLRs express in the synovial joints and cause inflammation that leads to swollen joints, pain, stiffness, and damage in cartilages and bones [44] (Figure 1). RA increases TNF-α and IL-1β in blood and synovial tissues that accelerate activation of matrix metalloproteinase (MMP) enzymes (e.g., MMP-1, MMP-9, and MMP-13) that can decompose all components of extracellular matrix and cartilages of joints [45,46]. Early diagnosis and treatment are essential to managing RA as psoriasis and bone damage are irreversible [34].

Several genetic, environmental, and socioeconomic factors are associated with RA initiation and progression [44]. Fat-rich and low-vegetable diet, smoking, gut microbiota, periodontal diseases, bacterial or viral infections lead to different inflammatory conditions and results in RA [47,48,49] (Figure 1). RA is also influenced by gender as women are more susceptible to RA than men and several factors influence the disease, such as hormonal deficiency (estrogen deficiency in pre- and post-menopausal women), genetic factor (X-chromosome related), the prevalence of depression, neuropathic pain, fibromyalgia and osteoporosis (in post-menopausal women) [50,51,52,53]. Apart from environmental factors, increased citrullination is also observed in alveolar macrophages in smokers’ lungs, and thus smoking may involve the pathogenesis of RA [47].

Citrullination is a peptidylarginine deiminase-mediated enzymatic conversion of arginine residues of protein to citrulline residues, and dysregulation of the peptidylarginine deiminases enzymes causes increased citrullination of protein in the joints of RA patients [54].

## 3. RA and Gastrointestinal Tract

The gastrointestinal tract is connected with the environment, and it is an essential getaway for the intake of beneficial and harmful microbes and their metabolites through food and drinks. The human gut harbors over 100 trillion bacteria, and these microbes have a potential role in the digestion, metabolism, nutrition, disease control, and maintenance of general well-being [55,56,57,58]. The epithelial cell barrier and intestinal single cell layer determine the rate of entry of microbes or antigens into the bloodstream [59,60]. Disturbance in the gastrointestinal homeostasis, especially massive changes in gut microbiota composition, results in diarrhea, dysentery, or inflammatory responses such as RA [61,62,63,64]. Increased bacterial lipopolysaccharides in the bloodstream and their deposition in the synovial fluids can cause proinflammatory responses (by releasing cytokines) and RA [65,66] (Figure 1).

Besides genetic, environmental, and physiological changes like citrullination of proteins and deposition in the joint tissues, RA is also believed to be caused by gut bacteria like *Prevotella. Prevotella* spp. are anaerobic, non-spore-forming bacteria, and these are part of normal gut flora. *Prevotella* contributes to polysaccharides breakdown, and their presence is relevant to the consumption of carbohydrate and high fiber diets [48]. *P. copri* is also responsible for the generation and progression of intestinal dysbiosis in people at their early stages of RA or risk on RA development [67,68,69]. Increased *Prevotella* spp. was found in the intestines of people carrying genotype of RA before developing clinical symptoms of the disease [70]. Increased abundance of *P. copri* and its antibodies are detected in patients with RA [71]. The increased population of *P. copri* in the intestinal lumen probably enables it to defend itself against myeloid cells and T cells that leads to immune responses [69] (Figure 1). *Prevotella* also activates the TLR-2 receptor of the intestinal epithelial cells and stimulates the release of proinflammatory cytokines such as IL-1β, IL-6, and IL-23 and promotes the activation of Th17 cells that leads to massive production of IL-17, inflammation, and initiation of RA [72] (Figure 1).

A meta-analysis study showed that probiotic supplement helps reduce IL-6 levels in RA patients, which may help to manage RA. However, it did not reduce DAS scores of RA patients, and it may not be effective in established RA patients [73]. It is known that gut dysbiosis favors increased population growth of *Eggerthella lenta* or *Collinsella aerofaciens* bacteria in the gut [74]. A clinical study showed decreased population of *Faecalibacterium* (a *Firmicutes* bacteria abundant in healthy human gut) in people experiencing RA [75] (Table 1). *Faecalibacterium* is responsible for butyrate production [76] that stimulates mucin secretion and lubrication of inner gut epithelium. As the *Faecalibacterium* population decreases, the gut is more vulnerable to opportunistic organisms like *Collinsella* and *Eggerthella. Collinsella* induces the release of cytokines (e.g., IL-17α) and chemokines (e.g., CXCL1 and CXCL5) that contribute to RA development by activating NFkB and neutrophils [75]. *Eggerthella* causes the citrullination of proteins that contributes to RA development. RA-associated decreased population of *Streptococcus* and *Haemophilus* are also observed in the human gut with the escalation of *Prevotella histicola* and *P. oulorum* numbers [77]. Higher fecal *Lactobacillus casei* was also reported in RA patients [78]. Thus dysbiosis-related changes in *Collinsella aerofaciens, Eggerthella lenta*, *Faecalibacterium* spp., *Haemophilus* spp., *Prevotella* spp., and *Streptococcus* spp. population induce loss of integrity of inner epithelium of gut and development of RA [75,77] (Table 1).

The situation regarding the enteric biome is complicated by the presence of pathogenic and commensal enteric viruses (forming the enteric virome) and their effects on intestinal homeostasis and immune responses. Since the subject matter is still challenging like experimenting with mice infected with commensal enteric human viruses is still not a feasible experiment that has been done [94], and since existing literature lacks reports (to our knowledge) on any enteric viral biome effect on initiation and development of RA, this review will confine itself to enteric microbial biome (meaning enteric bacteria and does not take into account virome or fungal biome) and any disturbances in their homeostasis leading to RA. The main objective of this review is to take a close look at whether the administration of probiotics in the form of *Lactobacillus casei* and *Lactobacillus acidophilus* can cause amelioration of the RA. However, it is possible that disturbances in the pattern recognition receptors (PRRs) and their signaling pathways can lead to disruption of immune homeostasis, and which might act as a trigger for RA.

It has been reported that the virome can have a profound influence on the composition and functional properties of the microbial biome, and in the practical sense, it alters the development and function of the immune system [95]. It remains for future studies to determine whether the predominance of *Prevotella* spp. in the human gut leading to RA is a result of gut virome alterations and whether taking probiotics like Lactobacillus can help the intestine to revert back to normal virome homeostasis. The question of gut inflammation, immunity, and the role of gut virome has been raised before [96], but any answer is yet to be found, and the topic falls outside the scope of this review. A very recent pre-print article reports dysbiotic oral and gut viromes in treated and un-treated RA patients [97]. Despite recent advances on gut virome and resultant pathogenesis if the homeostasis is disturbed, there is virtually no information on the role of gut virome dysbiosis and RA or even gut virome dysbiosis and consequential changes in the gut microbiome with *Prevotella copri* becoming the dominant oral and gut bacteria. It is left for scientists to discover to what extent gut microbiome and gut virome follow independent trajectories or have interacting trajectories; if interacting, when and where are the points of interaction?

## 4. *Prevotella* and RA Pathogenesis

Most likely, *P. copri* enter the bloodstream and infiltrate joints via phagocytic cells (such as macrophages, neutrophils, and dendritic cells) [71]. *P. copri* infection causes massive production of immunogenic helper T cell (Th17) cells, proinflammatory cytokines (such as IL-6, IL-17, and IL-23), leading to inflammation in the gut and that subsequently migrates to other organs and initiates RA as shown in a preclinical study [69,72]. An increased number of *P. copri* in the large intestine causes inflammatory responses in the gut. Th17 cells, IL-17, bacterial DNA, and possibly *Prevotella spp* migrate to the joints and induce joint inflammation and RA [79,80,81] (Figure 1). *Prevotella intermedia*, (another *Prevotella* bacteria) has been shown to increase prostaglandin E2 from arachidonic acid by activating cyclooxygenase-2 in the joints [98,99]. Noticeably, overexpression of the cyclooxygenase-2 enzyme was observed in RA [100]. Increased prostaglandins have multiple roles in RA pathogenesis, such as pain in the joints, inflammation, bone metabolism, and immune response [99].

*Prevotella* is also present in the oral cavity, and their presence increases in periodontal diseases, and thus RA is also correlated with periodontitis [49,101,102]. Periodontitis is highly correlated with RA in the clinic, and the oral bacterial population shift towards *Porphyromonas gingivalis* and other anaerobic bacteria such as *Prevotella spp.* [91,103] (Table 1). A concomitant increase in *Porphyromonas gingivalis* in the oral cavity and *P. copri* in the intestine is observed in patients who experience RA [92] (Figure 1). Serum samples from patients with RA showed positive responses to immunoglobulin G (IgG) antibody of *P. gingivalis* [104]. *P. gingivalis* from the oral cavity possibly migrate to the synovial tissues through phagocytotic capture of dendritic cells, and *P. gingivalis* also causes citrullination of proteins in synovial tissues, which in turn, produce systemic inflammatory responses [91,93].

Besides these, *P. histicola* and *P. intestinalis* have shown immunomodulatory effects and reduced arthritis in mice similar to RA [82,83,84]. *Prevotella intestinalis* may cause a reduction in short-chain fatty acids and IL-18 production in the intestine, which reduces the abundance of acetate-producing bacteria like *P. copri* [82]. *P. histicola* protects the intestinal mucosal barrier by increased enzymatic expression of antimicrobial compounds. The bacteria also produce anti-inflammatory effects by reducing inflammatory responses of Th17 cells and increasing the number of regulatory T cells and IL-10 [83] (Table 1).

## 5. Roles of *Lactobacillus* Probiotics against RA in Preclinical Studies

Probiotics are live microorganisms intended to be administered orally to improve the host’s gut microbiota [105,106]. Human gut microbiota sometimes becomes imbalanced and causes dysbiosis in situations like long-term treatment with antibiotics, NSAIDs, stress, and inflammatory conditions such as RA and osteoarthritis [107,108,109]. Bacteria like *Lactobacillus spp.* and *Bifidobacterium spp*. are widely used probiotics, and *Lactobacillus spp* can survive in an acidic gastrointestinal environment, and the presence of glucose in the gut help their survival [106,110]. The efficacy of probiotics depends on the microbial strain or pathophysiologic conditions of the host [109].

Treatment with *L. casei* or *L. acidophilus* over a period of 28 days reportedly prevented the development of arthritis in a preclinical model that reduced arthritic scores and proinflammatory cytokines such as IL-17, IL-1β, IL-6, and TNF-α, similar to that of indomethacin treatment [38]. The treatment with the *Lactobacillus spp* also increased the release of anti-inflammatory cytokines like IL-4 and IL-10 in the body fluids [38]. Consumption of *L. acidophilus* and *L. casei* reduces the oxidative stress of animals with collagen-induced arthritis [38]. Oral *L. casei* treatment also induced anti-inflammatory effects in rats in a collagen-induced arthritis model by inhibiting the COX-2 enzyme by reducing the proinflammatory cytokines [111]. Another preclinical study showed that intragastric administration of *L. casei* prevented the development of *Salmonella enterocolitis*-induced arthritis and reduced expression of proinflammatory cytokines (e.g., IL-1β, IL-6, IL-17, IL-23, and TNF-α) [85]. Treatment of *L. casei* at the initial stage of adjuvant-induced arthritis (AIA) model (similar to RA) in rats inhibited the development of arthritis that was comparable to methotrexate with normalization of gut microbiota and an increment of *L. acidophilus* population [86]. *L. acidophilus* treatment showed anti-inflammatory properties as it suppressed Th17 cell-mediated secretion of proinflammatory cytokines (IL-1β, IL-6, TNF-α, IL-17, and IL-23), but increased secretion of the anti-inflammatory cytokine (IL-10) [87,88]. Another study showed similar changes in the profile of cytokines along with reduced swelling, cartilage damage and lymphocyte infiltration in joints after *L. casei* treatment in collagen-induced arthritic rats [89].

Several *Lactobacillus* spp., especially *L. casei, L. reuteri, L. fermentum* and *L. rhamnosus* reduced collagen-induced-arthritis (CIA) in female Wistar rats by modifying gut microbial population (increasing *Lactobacillus* spp.), preventing proinflammatory cytokines, releasing antibodies, antibiotic substances and short-chain fatty acids, or modulating Th1/Th17 responses [90]. Noticeably, these effects are species dependent. *L. acidophilus* produces antibacterial substances, which are effective against in vitro Gram-positive and Gram-negative pathogenic bacteria, but not effective against normal gut flora [112,113].

On the negative side, *L. casei* [114], *L. bifidus L. salivarius, L. iners,* and *L. ruminis* are connected with pathogenesis of RA and increased populations of these bacteria have been observed in RA patients in comparison to healthy people [78]. Another study also showed that oral supplementation of *L.casei* and *L. acidophilus* increased phagocytic and lymphocytic activity in the intestinal mucosa of Swiss albino mice [115]. Therefore, the consumption of *L.casei* and *L. acidophilus* can sometimes but rarely produce harmful effects, which is probably due to the increased population of these bacteria in the human gut. There are variations of effects between *Lactobacillus* spp., treatment protocols and induction of experimental rheumatoid arthritis, animal species, and measurement parameters as reported previously [78]. Therefore, the outcome of these studies is quite inconclusive, but we hypothesize that a moderate population of *L. casei* and *L. acidophilus* produces beneficial effects. However, an increased population may cause infiltration of these bacteria from the gut to other organs, thereby producing harmful effects.

## 6. Roles of *Lactobacillus* Probiotics against RA in the Clinic

Patients with RA showed an increased presence of *Bacteroides, Escherichia*, and *Shigella* bacteria in the gut with a marked decrease in *Lactobacillus* spp. [116]. A well-balanced gut microflora provides essential vitamins like B-vitamins B3, B5, B6 (pyridoxal phosphate), B7, and B12, folate, tetrahydrofolate, and vitamin-K [58]. Low plasma vitamin-B6 has been observed in inflammatory conditions like RA, and long-term treatment with NSAIDs such as cyclooxygenase inhibitors prevented vitamin-B6 metabolism and thus reduced pyridoxal phosphate concentration from blood [117]. Impaired secretion of these essential vitamins (in conditions like gut dysbiosis), especially vitamin-B6 deficiency, can cause RA and cardiovascular complications [118,119]. Adequate colonization of *Lactobacillus spp* can improve the epithelium’s integrity, and it becomes less susceptible to *Bacteroides, Escherichia*, and *Shigella* infections and their translocation into the intestinal lumen [120,121,122,123]. The bacteria secrete multiple short-chain fatty acids (e.g., lactic, acetic, and polyglutamic acid) and vitamins that provide nutritional support and decrease the pH of the intestinal lumen that prevent the colonization of harmful bacteria [124,125,126].

*L. casei* treatment over a period of 8 weeks improved RA-related pathophysiological parameters in a randomized clinical trial [127]. *Lactobacillus spp* also acts as an antimicrobial agent against different microorganisms [124,128]. Patients with RA show increased nitric oxide and reactive oxygenated species in blood and synovium, which cause degradation of lipids, other macromolecules, and matrix of the affected person [129,130].

Once daily administration of *L. casei* in capsule (contained 10^8^ colony-forming unit) consumption treatment over a period of 8 weeks reduced the swelling of joints, arthritis-related disease activity score-28, serum high-sensitivity C-reactive protein (hs-CRP) levels, and proinflammatory cytokines, especially TNF-α and IL-12 in women with RA relative to the placebo-treated control group. *L. casei* increased plasma IL-10 levels after the treatment but caused no changes in IL-1β and IL-6 levels [131]. Similarly, another study with a similar *L. casei* treatment protocol on a smaller number of patients found similar changes in the cytokines profile of RA patients [132]. The same dose of *L. casei* administered over eight weeks did not change the oxidative stress indicators or lipid profiles of RA patients [133,134]. Regular consumption of *L. casei* preserves the gastrointestinal diversity and prevents gastrointestinal dysbiosis, physiological stress, RA, and other inflammatory disorders [107]. *L. acidophilus* supplements in diabetic patients (*n* = 48, *p*= 24, c = 24) over a period of 4 weeks did not modify systemic inflammatory responses (induced by *Escherichia coli* lipopolysaccharide injections) and insulin sensitivity [135] (Table 2). The randomized clinical trials of probiotics are not readily comparable among these, as there are variations in the patient selection, probiotic formulation, experimental parameters, dose, and frequencies of probiotic treatment, and all these can potentially affect the experimental outcomes (Table 2).

## 7. Possible Effector Molecules of *Lactobacillus* Species

Intestinal microbes and their gene products can interact with PRRs, including *C*-type lectin receptors, including the specific intracellular adhesion molecule-3 grabbing non-integrin homolog-related 3 (SIGNR3) [140,141]. Any disruption of the delicate balance between the intestinal biome and their various gene product interactions can lead to disruptions in signal-transducing, giving rise to diseases like irritable bowel syndrome [142]. *Lactobacillus acidophilus* NCFM has been described as a model probiotic strain [143]. The strain is distinguished by its several surface (S) layer proteins. The S layer is encoded by three Slp-encoding genes: slpA (LBA0169), slpB (LBA0175), and slpX (LBA0512). SlpA, a probiotic factor, can bind to the host immune receptor SIGNR3, a *C*-type lectin. The binding affects modulatory signals, resulting in the mitigation of colitis, maintenance of a healthy gut biome, and protection of gut mucosal barrier function [143]. Other documented effector molecules (besides SlpA) in various species of *Lactobacillus* and *Bifidobacterium* strains have been reviewed by Lebeer and colleagues [143]. These molecules include specific pili (*Lactobacillus rhamnosus* GG), S-layer proteins (*Lactobacillus acidophilus* NCFM), exopolysaccharides and muropeptides. Various metabolites like tryptophan- and histamine-related metabolites (*Lactobacillus reuteri* 6475), CpG-rich DNA, and enzymes like lactase and bile salt hydrolases can also act as effector molecules.

*Lactobacillus*, often neglected because of the notion that they are transient passengers of the gut [144], now are regarded as important members of the gut microbiome, because they can form up to 5% of the gut microbiota and up to 99% of vaginal microbiota [145]. However, not much study is available on the effector molecule(s) of *Lactobaccilus casei* and *Lactobacillus acidophilus* that may play a significant role in the attenuation of RA. Consumption of *Lactobacillus casei* fermented milk reportedly prevented *Salmonella enteritidis* reactive arthritis [85]. Such prevention occurred through modulation of IL23/IL17 expression. On the other hand, adhesion of probiotic bacteria to the gastrointestinal surface is considered a prerequisite for the exclusion of competitive pathogens [146], which would suggest that *Lactobacillus casei* adhesion molecules like fucose may play the part of an effector molecule [85].

## 8. Conclusions

In summary, preclinical studies mainly focus on the preventative roles of *Lactobacillus spp* in various experimental models of RA. In contrast, several clinical studies have investigated this bacteria’s role in the improvement of the symptoms and diagnostic parameters after RA establishment. *L. casei* or *L. acidophilus* supplementation (in the form of food, drink, or pharmaceutical dosage forms) may not have an immediate favorable impact against RA. It ameliorates gut dysbiosis and consequential RA pathogenesis after repeated long-term use. However, an increased population of *Lactobacillus salivarius* was recorded in the gut, teeth and saliva of RA patients and *L. salivarius* and some other *Lactobacillus* spp. could be associated with RA pathogenesis [145,147]. Long-term high-dose consumption of some *Lactobacillus* spp. may induce rare complications like liver abscess and bacteremia [148,149,150]. Therefore, the benefits of *Lactobacillus* probiotic supplements depend on the *Lactobacillus* species. Since *L. casei* and *L. acidophilus* have anti-inflammatory, antimicrobial, antioxidant properties, these bacteria act symbiotically in the gut to establish their colonization and thus increase the integrity of cellular layers (of the gastrointestinal tract), maintain nutritional support of the host, and reduce the severity of inflammatory conditions like RA.

It has been put forward that the therapeutic use of probiotics and prebiotics in the case of obesity-related non-alcoholic fatty liver disease did not demonstrate any major benefits in high-quality clinical trial studies [151]. A number of factors are involved in probiotic studies, namely the initial composition of the patient’s gut and possibly oral microbiome, the nature of probiotic administered, the progress of the disease at the time of probiotic administration, and the continuation of the diet and the probiotic along with any other medications. Every disease possibly has its own probiotics regimen and the initial microbiome of the patient, which brings in not one but two factors. As such, probiotics can be and more possibly should be regarded as nutraceutical supplements, and it will be somewhat futile to presume that what will not work for obesity-related non-alcoholic fatty liver disease will also not work for, say RA.

Probiotics can be used as nutritional supplements to manage inflammatory disorders like rheumatoid arthritis, diarrhea, dysentery, irritable bowel syndrome, diabetes, but the effectiveness in clinical trials is yet to be proved. Probiotic supplements should be considered as a functional food which has some therapeutic benefits. The quality of probiotic products is currently examined based on the number of viable colonies of certain probiotic bacteria (e.g., *L. acidophilus)* in the product [152]. However, the number of colonies cannot represent the effectiveness of the bacteria for inflammatory disorders like RA. Physical parameters, such as stability in a low pH environment (e.g., gastric lumen), physical stability, viability and proper storage condition, should be monitored to maintain good quality products [153]. In vitro laboratory assessments like adhesion capabilities of probiotic bacteria to human intestinal epithelial cells (such as Caco-2 cell line) and the capability to contribute to the lubrication of inner gut walls (in vivo) needs to be taken into account [152,154,155]. New quality control parameters should be implemented to maintain an optimum standard of probiotic products for consumers.

## Figures and Tables

**Table 1:** The roles of some bacteria in RA pathogenesis.

Bacteria	Changes During Gut Dysbiosis	Roles in RA Pathogenesis or Prevention	Ref.
***Collinsella***	Increased population	reduces expression of tight junction protein in gut epithelial cells increases gut permeability releases proinflammatory cytokines (e.g., IL-17α) secretes chemokines (e.g., CXCL1 and CXCL5) cytokines and chemokines activate NFkB and neutrophils Inflammation in gut epithelium and RA development	[75]
***Eggerthella***	Increased population	conversion of arginine residues of protein to citrulline residues increases citrullination of proteins in gut citrullination contributes to RA development	[75]
***Faecalibacterium***	Decreased population	responsible for butyrate production butyrate stimulates mucin secretion and lubrication of gut epithelium reduced *Faecalibacterium*, reduces lubrication on epithelium and makes it vulnerable to pathogens reduced *Faecalibacterium* provides increased growth of *Collinsella and Eggerthella*	[76]
***Prevotella***	Increased population	*Prevotella spp.* contributes to polysaccharides breakdown *P. copri* acts against immune responses of T-cells *P. copri* activates TLR-2 receptor of intestinal epithelial cells *P. copri* stimulate secretions of IL-1β, IL-6, IL-17, and IL-23 *P. copri* causes inflammation in GI lumen and initiates RA *P. histicola* releases antimicrobial compounds *P. histicola* and *P. intestinalis* have anti-inflammatory effects *P. histicola* increases expression of Treg cells and IL-10 Some effects are species-dependent	[69,72,79,80,81,82,83,84]
***Lactobacillus***	Decreased population	reduces proinflammatory cytokines (e.g., IL-17, IL-1β, IL-6, and TNF-α) releases anti-inflammatory cytokines like IL-4 and IL-10 reduces oxidative stress reduces swelling and cartilage damages reduces lymphocyte infiltration in joints releases short-chain fatty acids reduces growth *of Bacteroides, Escherichia* and *Shigella* in gut some effects are species dependent	[38,85,86,87,88,89,90]
***Porphyromonas*** ***gingivalis***	Increased population	oral *P. gingivalis* increases with concomitant growth of intestinal *P. copri* *P. gingivalis* possibly migrates to synovial tissues causes citrullination of proteins in synovial tissues produces inflammatory responses and RA	[91,92,93]

**Table 2:** Effects of probiotics in clinical trials.

Sample Size	Probiotic Type, Control and Duration	Measurement Parameters	Brief Outcome	Ref.
RCT, 54 (C: 27, P:27)	P: *L. acidophilus* (2 × 10^9^ cfu/g), *L. casei* (2 × 10^9^ cfu/g), *B. bifidum* (2 × 10^9^ cfu/g), 0.8 g inulin C: starch Duration: 8 weeks, Dose: 1 cap/day	DAS-28, hs-CRP, VAS, NO, insulin levels, HOMA-IR, HOMA-B, GSH levels	Improved: hs-CRP, DAS-28, VAS, NO, insulin levels, HOMA-IR, HOMA-B, and GSH levels	[136]
RCT, 60 (C: 30, P: 30)	P: *L. acidophilus* (2 × 10^9^ cfu/g), *L. casei* (2 × 10^9^ cfu/g), *B. bifidum* (2 × 10^9^ cfu/g) C: cellulose, Duration: 8 weeks, Dose: 1 cap/day	DAS-28, HOMA-B, hs-CRP, insulin levels	Improved: DAS-28, Decreased: insulin, HOMA-B, and hs-CRP levels	[127]
RCT, 46 (C: 24, P: 22)	P: *L. casei* 01 (10^8^ cfu), C: maltodextrin Duration: 8 weeks Dose: 1 cap/day	MDA, TAC, SOD, GPx, CAT	No changes observed.	[133]
RCT, 46, (C: 24, P: 22)	P: *L. casei* 01 (10^8^ cfu), C: maltodextrin Duration: 8 weeks Dose: 1 cap/day	DAS28, serum cytokines (IL-1β, IL-6, IL-10, IL-12 and TNF-α), EULAR	Increased: IL-10, Reduced: TNF- α and IL-12	[131]
RCT, 46 (C: 24, P: 22)	P: *L. casei* 01 (10^8^ cfu), C: maltodextrin Duration: 8 weeks Dose: 1 cap/day	Cytokines (TNF-α, IL-6, IL-12)	Increased: IL-10, IL-10:IL-12 ratio. Reduced: TNF- α, IL-6 and IL-12	[132]
RCT, 29 (C: 14, P: 15)	P: *L. rhamnosus* GR-1, *L. reuteri* RC-14 (total: 2 × 10^9^ cfu) C: dextrose, starch, mcc, magnesium stearate Duration: 3 months Dose: 2 caps/day	ACR20 responses, cytokine levels	No changes observed.	[137]
RCT, 44 (C: 22, P: 22)	P: *Bacillus coagulans* GBI-30,6086, green tea extract, msm, vitamins (A, B, C, D, E), Se, C: mcc Duration: 2 months Dose: 1 cap/day	ACR, HAQ-DI, ESR, CRP	Reduced: CRP, and pain scores	[138]
RCT, 21 (C: 13, P: 8)	P: *L. rhamnosus* GG (5 × 10^9^ cfu), C: placebo (no info), Duration: 12 months Dose: 4 caps/day	RA activity, HAQ index, CRP, ESR cytokines (IL-6, TNF-α, MPO, IL-10 and IL-12)	No changes observed.	[139]
RCT, 48 (C: 24, P: 24)	P: *L. acidophilus* NCFM (1 g, 10^10^ cfu), C: placebo (silicium dioxide and lactose, 1:1), Dose:	Systemic inflammatory response (by *E. coli* LPS), insulin resistance	No changes observed	[135]

RCT, randomized control trial; C, control (or placebo) group; P, probiotic (treatment) group; B., *Bifidobacterium*; *L., Lactobacillus*; DAS-28, Disease Activity Score of 28 joints; ACR, American College of Rheumatology criteria (e.g., ACR20), HOMA-B, homeostatic model assessment-B homoeostatic model assessment-β-cell function; hs-CRP, serum high-sensitivity C-reactive protein; HOMA-IR, homoeostasis model of assessment estimated insulin resistance; RA, rheumatoid arthritis; VAS, visual analogue scale of pain; NO, nitric oxide; MDA, serum malondialdehyde; TAC, total antioxidant capacity; SOD, erythrocyte superoxide dismutase; GPx, glutathione peroxidase; CAT, catalase; EULAR, European League Against Rheumatism response; HAQ-DI, Stanford Health Assessment Questionnaire Disability Index, mcc, microcrystalline cellulose; ESR, erythrocyte sedimentation rate; CRP, C-reactive protein; HAQ, Health Assessment Questionnaire; MPO, myeloperoxidase; Se, selenium; LPS: lipopolysaccharides; FA, folic acid; msm, methylsulfonylmethane.

## References

[1] NIAMS National Institute of Arthritis and Musculoskeletal and Skin Diseases. https://www.niams.nih.gov/health-topics/rheumatoid-arthritis.

[2] Tak P.P., Bresnihan B. (2000). The pathogenesis and prevention of joint damage in rheumatoid arthritis: Advances from synovial biopsy and tissue analysis. Arthritis Rheum..

[3] WHO Chronic Rheumatic Conditions—WHO. https://www.who.int/chp/topics/rheumatic/en/.

[4] AIHW, Australian Institute of Health Welfare Rheumatoid Arthritis. https://www.aihw.gov.au/reports/chronic-musculoskeletal-conditions/rheumatoid-arthritis.

[5] Lassere M.N., Rappo J., Portek I.J., Sturgess A., Edmonds J.P. (2013). How many life years are lost in patients with rheumatoid arthritis? Secular cause-specific and all-cause mortality in rheumatoid arthritis, and their predictors in a long-term Australian cohort study. Intern. Med. J..

[6] Therapeutic Guidelines Principles of nonsteroidal anti-inflammatory drug use for musculoskeletal conditions in adults. eTG complete Melbourne: Therapeutic Guidelines Limited.

[7] Wang W., Zhou H., Liu L. (2018). Side effects of methotrexate therapy for rheumatoid arthritis: A systematic review. Eur. J. Med. Chem..

[8] Tilstra J.S., Lienesch D.W. (2015). Rheumatoid Nodules. Dermatol. Clin..

[9] Sepriano A., Kerschbaumer A., Smolen J.S., van der Heijde D., Dougados M., van Vollenhoven R., McInnes I.B., Bijlsma J.W., Burmester G.R., de Wit M. (2020). Safety of synthetic and biological DMARDs: A systematic literature review informing the 2019 update of the EULAR recommendations for the management of rheumatoid arthritis. Ann. Rheum. Dis..

[10] Schrezenmeier E., Dörner T. (2020). Mechanisms of action of hydroxychloroquine and chloroquine: Implications for rheumatology. Nat. Rev. Rheumatol..

[11] Dos Reis Neto E.T., Kakehasi A.M., de Medeiros Pinheiro M., Ferreira G.A., Marques C.D.L., da Mota L.M.H., Dos Santos Paiva E., Pileggi G.C.S., Sato E.I., Reis A. (2020). Revisiting hydroxychloroquine and chloroquine for patients with chronic immunity-mediated inflammatory rheumatic diseases. Adv. Rheumatol..

[12] Jorge A., Ung C., Young L.H., Melles R.B., Choi H.K. (2018). Hydroxychloroquine retinopathy—Implications of research advances for rheumatology care. Nat. Rev. Rheumatol..

[13] Stokkermans T.J., Goyal A., Bansal P., Trichonas G. (2020). Chloroquine and Hydroxychloroquine Toxicity. StatPearls (Internet).

[14] Gerriets V., Bansal P., Goyal A., Khaddour K. (2020). Tumor Necrosis Factor Inhibitors. StatPearls (Internet).

[15] Zelová H., Hošek J. (2013). TNF-α signalling and inflammation: Interactions between old acquaintances. Inflamm. Res..

[16] Emery P., Vlahos B., Szczypa P., Thakur M., Jones H.E., Woolcott J., Santos Estrella P.V., Rolland C., Gibofsky A., Citera G. (2020). Longterm Drug Survival of Tumor Necrosis Factor Inhibitors in Patients with Rheumatoid Arthritis. J. Rheumatol..

[17] Reed G.W., Gerber R.A., Shan Y., Takiya L., Dandreo K.J., Gruben D., Kremer J., Wallenstein G. (2019). Real-World Comparative Effectiveness of Tofacitinib and Tumor Necrosis Factor Inhibitors as Monotherapy and Combination Therapy for Treatment of Rheumatoid Arthritis. Rheumatol. Ther..

[18] Furst D.E. (2010). The risk of infections with biologic therapies for rheumatoid arthritis. Semin. Arthritis Rheum..

[19] Pérez-Sola M.J., Torre-Cisneros J., Pérez-Zafrilla B., Carmona L., Descalzo M.A., Gómez-Reino J.J. (2011). Infections in patients treated with tumor necrosis factor antagonists: Incidence, etiology and mortality in the BIOBADASER registry. Med. Clin..

[20] Lindhaus C., Tittelbach J., Elsner P. (2017). Cutaneous side effects of TNF-alpha inhibitors. J. Dtsch. Dermatol. Ges..

[21] Krathen M.S., Gottlieb A.B., Mease P.J. (2010). Pharmacologic immunomodulation and cutaneous malignancy in rheumatoid arthritis, psoriasis, and psoriatic arthritis. J. Rheumatol..

[22] Chen Y., Friedman M., Liu G., Deodhar A., Chu C.Q. (2018). Do tumor necrosis factor inhibitors increase cancer risk in patients with chronic immune-mediated inflammatory disorders?. Cytokine.

[23] Raaschou P., Söderling J., Turesson C., Askling J. (2018). Tumor necrosis factor inhibitors and cancer recurrence in Swedish patients with rheumatoid arthritis: A nationwide population-based cohort study. Ann. Intern. Med..

[24] Malysheva O., Baerwald C.G. (2011). Low-dose corticosteroids and disease modifying drugs in patients with rheumatoid arthritis. Clin. Exp. Rheumatol..

[25] Petta I., Peene I., Elewaut D., Vereecke L., De Bosscher K. (2019). Risks and benefits of corticosteroids in arthritic diseases in the clinic. Biochem. Pharmacol..

[26] Aletaha D., Smolen J.S. (2018). Diagnosis and management of rheumatoid arthritis: A review. JAMA.

[27] Cruz-Topete D., Cidlowski J.A. (2015). One hormone, two actions: Anti-and proinflammatory effects of glucocorticoids. Neuroimmunomodulation.

[28] Sorrells S.F., Sapolsky R.M. (2007). An inflammatory review of glucocorticoid actions in the CNS. Brain Behav. Immun..

[29] Park J.H., Lee N.K., Lee S.Y. (2017). Current Understanding of RANK Signaling in Osteoclast Differentiation and Maturation. Mol. Cells.

[30] Hatano Y., Matsuoka H., Lam L., Currow D.C. (2018). Side effects of corticosteroids in patients with advanced cancer: A systematic review. Support. Care Cancer.

[31] Mateen S., Zafar A., Moin S., Khan A.Q., Zubair S. (2016). Understanding the role of cytokines in the pathogenesis of rheumatoid arthritis. Clin. Chim. Acta.

[32] Arend W.P., Dayer J.M. (1990). Cytokines and cytokine inhibitors or antagonists in rheumatoid arthritis. Arthritis Rheum..

[33] Xu L., Feng X., Tan W., Gu W., Guo D., Zhang M., Wang F. (2013). IL-29 enhances Toll-like receptor-mediated IL-6 and IL-8 production by the synovial fibroblasts from rheumatoid arthritis patients. Arthritis Res. Ther..

[34] Thompson C., Davies R., Choy E. (2016). Anti cytokine therapy in chronic inflammatory arthritis. Cytokine.

[35] Kishimoto T. (2019). Discovery of IL-6 and development of anti-IL-6R antibody. Keio J. Med..

[36] Alam J., Jantan I., Bukhari S.N.A. (2017). Rheumatoid arthritis: Recent advances on its etiology, role of cytokines and pharmacotherapy. Biomed. Pharmacother..

[37] Pradhan A., Bagchi A., De S., Mitra S., Mukherjee S., Ghosh P., Ghosh A., Chatterjee M. (2019). Role of redox imbalance and cytokines in mediating oxidative damage and disease progression of patients with rheumatoid arthritis. Free Radic. Res..

[38] Amdekar S., Singh V., Kumar A., Sharma P., Singh R. (2013). *Lactobacillus casei* and *Lactobacillus acidophilus* regulate inflammatory pathway and improve antioxidant status in collagen-induced arthritic rats. J. Interferon Cytokine Res..

[39] van Hamburg J.P., Tas S.W. (2018). Molecular mechanisms underpinning T helper 17 cell heterogeneity and functions in rheumatoid arthritis. J. Autoimmun..

[40] Chen J.Q., Szodoray P., Zeher M. (2016). Toll-Like Receptor Pathways in Autoimmune Diseases. Clin. Rev. Allergy Immunol..

[41] Piccinini A.M., Williams L., McCann F.E., Midwood K.S. (2016). Investigating the role of Toll-like receptors in models of arthritis. Methods Mol. Biol..

[42] Elshabrawy H.A., Essani A.E., Szekanecz Z., Fox D.A., Shahrara S. (2017). TLRs, future potential therapeutic targets for RA. Autoimmun. Rev..

[43] McGarry T., Veale D.J., Gao W., Orr C., Fearon U., Connolly M. (2015). Toll-like receptor 2 (TLR2) induces migration and invasive mechanisms in rheumatoid arthritis. Arthritis Res. Ther..

[44] McInnes I.B., Schett G. (2011). The pathogenesis of rheumatoid arthritis. N. Engl. J. Med..

[45] Burrage P.S., Mix K.S., Brinckerhoff C.E. (2006). Matrix metalloproteinases: Role in arthritis. Front. Biosci..

[46] Malemud C.J. (2017). Matrix Metalloproteinases and Synovial Joint Pathology. Prog. Mol. Biol. Transl. Sci..

[47] Klareskog L., Stolt P., Lundberg K., Källberg H., Bengtsson C., Grunewald J., Rönnelid J., Harris H.E., Ulfgren A.K., Rantapää-Dahlqvist S. (2006). A new model for an etiology of rheumatoid arthritis: Smoking may trigger HLA-DR (shared epitope)-restricted immune reactions to autoantigens modified by citrullination. Arthritis Rheum..

[48] Precup G., Vodnar D.C. (2019). Gut Prevotella as a possible biomarker of diet and its eubiotic versus dysbiotic roles: A comprehensive literature review. Br. J. Nutr..

[49] Möller B., Kollert F., Sculean A., Villiger P.M. (2020). Infectious Triggers in Periodontitis and the Gut in Rheumatoid Arthritis (RA): A Complex Story About Association and Causality. Front. Immunol..

[50] van Vollenhoven R.F. (2009). Sex differences in rheumatoid arthritis: More than meets the eye. BMC Med..

[51] Sapir-Koren R., Livshits G. (2017). Postmenopausal osteoporosis in rheumatoid arthritis: The estrogen deficiency-immune mechanisms link. Bone.

[52] Favalli E.G., Biggioggero M., Crotti C., Becciolini A., Raimondo M.G., Meroni P.L. (2019). Sex and Management of Rheumatoid Arthritis. Clin. Rev. Allergy Immunol..

[53] Islander U., Jochems C., Lagerquist M.K., Forsblad-d’Elia H., Carlsten H. (2011). Estrogens in rheumatoid arthritis; the immune system and bone. Mol. Cell. Endocrinol..

[54] Fert-Bober J., Darrah E., Andrade F. (2020). Insights into the study and origin of the citrullinome in rheumatoid arthritis. Immunol. Rev..

[55] Ramezani A., Raj D.S. (2014). The gut microbiome, kidney disease, and targeted interventions. J. Am. Soc. Nephrol..

[56] Dias Bastos P.A., Lara Santos L., Pinheiro Vitorino R.M. (2018). How are the expression patterns of gut antimicrobial peptides modulated by human gastrointestinal diseases? A bridge between infectious, inflammatory, and malignant diseases. J. Pept. Sci..

[57] Boulangé C.L., Neves A.L., Chilloux J., Nicholson J.K., Dumas M.E. (2016). Impact of the gut microbiota on inflammation, obesity, and metabolic disease. Genome Med..

[58] Kau A.L., Ahern P.P., Griffin N.W., Goodman A.L., Gordon J.I. (2011). Human nutrition, the gut microbiome and the immune system. Nature.

[59] Wells J.M., Brummer R.J., Derrien M., MacDonald T.T., Troost F., Cani P.D., Theodorou V., Dekker J., Méheust A., de Vos W.M. (2017). Homeostasis of the gut barrier and potential biomarkers. Am. J. Physiol. Gastrointest. Liver Physiol..

[60] Haber A.L., Biton M., Rogel N., Herbst R.H., Shekhar K., Smillie C., Burgin G., Delorey T.M., Howitt M.R., Katz Y. (2017). A single-cell survey of the small intestinal epithelium. Nature.

[61] Bodkhe R., Balakrishnan B., Taneja V. (2019). The role of microbiome in rheumatoid arthritis treatment. Ther. Adv. Musculoskelet. Dis..

[62] Manasson J., Blank R.B., Scher J.U. (2020). The microbiome in rheumatology: Where are we and where should we go?. Ann. Rheum. Dis..

[63] Rouhani S., Griffin N.W., Yori P.P., Gehrig J.L., Olortegui M.P., Salas M.S., Trigoso D.R., Moulton L.H., Houpt E.R., Barratt M.J. (2019). Diarrhea as a Potential Cause and Consequence of Reduced Gut Microbial Diversity Among Undernourished Children in Peru. Clin. Infect. Dis..

[64] Paul A.K., Paul A., Jannat K., Afrose S., Bondhon T.A., Hasan A., Jahan R., Rahmatullah M. (2021). The Role of *Lactobacillus* Probiotics in Dysentery. EC Gastroenterol. Dig. Syst..

[65] Huang Z.Y., Stabler T., Pei F.X., Kraus V.B. (2016). Both systemic and local lipopolysaccharide (LPS) burden are associated with knee OA severity and inflammation. Osteoarthr. Cartil..

[66] Pretorius E., Akeredolu O.O., Soma P., Kell D.B. (2017). Major involvement of bacterial components in rheumatoid arthritis and its accompanying oxidative stress, systemic inflammation and hypercoagulability. Exp. Biol. Med..

[67] Alpizar-Rodriguez D., Lesker T.R., Gronow A., Gilbert B., Raemy E., Lamacchia C., Gabay C., Finckh A., Strowig T. (2019). *Prevotella copri* in individuals at risk for rheumatoid arthritis. Ann. Rheum. Dis..

[68] Scher J.U., Sczesnak A., Longman R.S., Segata N., Ubeda C., Bielski C., Rostron T., Cerundolo V., Pamer E.G., Abramson S.B. (2013). Expansion of intestinal *Prevotella copri* correlates with enhanced susceptibility to arthritis. eLife.

[69] Maeda Y., Kurakawa T., Umemoto E., Motooka D., Ito Y., Gotoh K., Hirota K., Matsushita M., Furuta Y., Narazaki M. (2016). dysbiosis Contributes to Arthritis Development via Activation of Autoreactive T Cells in the Intestine. Arthritis Rheumatol..

[70] Wells P.M., Adebayo A.S., Bowyer R.C.E., Freidin M.B., Finckh A., Strowig T., Lesker T.R., Alpizar-Rodriguez D., Gilbert B., Kirkham B. (2020). Associations between gut microbiota and genetic risk for rheumatoid arthritis in the absence of disease: A cross-sectional study. Lancet Rheumatol..

[71] Pianta A., Arvikar S., Strle K., Drouin E.E., Wang Q., Costello C.E., Steere A.C. (2017). Evidence of the Immune Relevance of *Prevotella copri*, a Gut Microbe, in Patients With Rheumatoid Arthritis. Arthritis Rheumatol..

[72] Larsen J.M. (2017). The immune response to *Prevotella* bacteria in chronic inflammatory disease. Immunology.

[73] Mohammed A.T., Khattab M., Ahmed A.M., Turk T., Sakr N., Khalil A.M., Abdelhalim M., Sawaf B., Hirayama K., Huy N.T. (2017). The therapeutic effect of probiotics on rheumatoid arthritis: A systematic review and meta-analysis of randomized control trials. Clin. Rheumatol..

[74] Balakrishnan B., Luckey D., Taneja V. (2019). Autoimmunity-associated gut commensals modulate gut permeability and immunity in humanized mice. Mil. Med..

[75] Chen J., Wright K., Davis J.M., Jeraldo P., Marietta E.V., Murray J., Nelson H., Matteson E.L., Taneja V. (2016). An expansion of rare lineage intestinal microbes characterizes rheumatoid arthritis. Genome Med..

[76] Ferreira-Halder C.V., Faria A.V.S., Andrade S.S. (2017). Action and function of *Faecalibacterium prausnitzii* in health and disease. Best Pract. Res. Clin. Gastroenterol..

[77] Chu X.J., Cao N.W., Zhou H.Y., Meng X., Guo B., Zhang H.Y., Li B.Z. (2021). The oral and gut microbiome in rheumatoid arthritis patients: A systematic review. Rheumatology.

[78] Liu X., Zou Q., Zeng B., Fang Y., Wei H. (2013). Analysis of fecal Lactobacillus community structure in patients with early rheumatoid arthritis. Curr. Microbiol..

[79] Martinez-Martinez R.E., Abud-Mendoza C., Patiño-Marin N., Rizo-Rodríguez J.C., Little J.W., Loyola-Rodríguez J.P. (2009). Detection of periodontal bacterial DNA in serum and synovial fluid in refractory rheumatoid arthritis patients. J. Clin. Periodontol..

[80] Reichert S., Haffner M., Keyßer G., Schäfer C., Stein J.M., Schaller H.G., Wienke A., Strauss H., Heide S., Schulz S. (2013). Detection of oral bacterial DNA in synovial fluid. J. Clin. Periodontol..

[81] Moen K., Brun J.G., Valen M., Skartveit L., Eribe E.K., Olsen I., Jonsson R. (2006). Synovial inflammation in active rheumatoid arthritis and psoriatic arthritis facilitates trapping of a variety of oral bacterial DNAs. Clin. Exp. Rheumatol..

[82] Iljazovic A., Roy U., Gálvez E.J.C., Lesker T.R., Zhao B., Gronow A., Amend L., Will S.E., Hofmann J.D., Pils M.C. (2021). Perturbation of the gut microbiome by *Prevotella* spp. enhances host susceptibility to mucosal inflammation. Mucosal Immunol..

[83] Marietta E.V., Murray J.A., Luckey D.H., Jeraldo P.R., Lamba A., Patel R., Luthra H.S., Mangalam A., Taneja V. (2016). Suppression of Inflammatory Arthritis by Human Gut-Derived *Prevotella histicola* in Humanized Mice. Arthritis Rheumatol..

[84] Maeda Y., Takeda K. (2017). Role of Gut Microbiota in Rheumatoid Arthritis. J. Clin. Med..

[85] Noto Llana M., Sarnacki S.H., Aya Castañeda Mdel R., Bernal M.I., Giacomodonato M.N., Cerquetti M.C. (2013). Consumption of *Lactobacillus casei* fermented milk prevents Salmonella reactive arthritis by modulating IL-23/IL-17 expression. PLoS ONE.

[86] Pan H., Guo R., Ju Y., Wang Q., Zhu J., Xie Y., Zheng Y., Li T., Liu Z., Lu L. (2019). A single bacterium restores the microbiome dysbiosis to protect bones from destruction in a rat model of rheumatoid arthritis. Microbiome.

[87] Chen L., Zou Y., Peng J., Lu F., Yin Y., Li F., Yang J. (2015). Lactobacillus acidophilus suppresses colitis-associated activation of the IL-23/Th17 axis. J. Immunol. Res..

[88] Park J.S., Choi J.W., Jhun J., Kwon J.Y., Lee B.I., Yang C.W., Park S.H., Cho M.L. (2018). *Lactobacillus acidophilus* Improves Intestinal Inflammation in an Acute Colitis Mouse Model by Regulation of Th17 and Treg Cell Balance and Fibrosis Development. J. Med. Food.

[89] So J.S., Lee C.G., Kwon H.K., Yi H.J., Chae C.S., Park J.A., Hwang K.C., Im S.H. (2008). *Lactobacillus casei* potentiates induction of oral tolerance in experimental arthritis. Mol. Immunol..

[90] Fan Z., Yang B., Ross R.P., Stanton C., Zhao J., Zhang H., Chen W. (2020). The prophylactic effects of different Lactobacilli on collagen-induced arthritis in rats. Food Funct..

[91] Jung H., Jung S.M., Rim Y.A., Park N., Nam Y., Lee J., Park S.H., Ju J.H. (2017). Arthritic role of *Porphyromonas gingivalis* in collagen-induced arthritis mice. PLoS ONE.

[92] Drago L., Zuccotti G.V., Romanò C.L., Goswami K., Villafañe J.H., Mattina R., Parvizi J. (2019). Oral-Gut Microbiota and Arthritis: Is There an Evidence-Based Axis?. J. Clin. Med..

[93] Carrion J., Scisci E., Miles B., Sabino G.J., Zeituni A.E., Gu Y., Bear A., Genco C.A., Brown D.L., Cutler C.W. (2012). Microbial carriage state of peripheral blood dendritic cells (DCs) in chronic periodontitis influences DC differentiation, atherogenic potential. J. Immunol..

[94] Metzger R.N., Krug A.B., Eisenächer K. (2018). Enteric virome sensing—Its role in intestinal homeostasis and immunity. Viruses.

[95] De Paepe M., Leclerc M., Tinsley C.R., Petit M.A. (2014). Bacteriophages: An underestimated role in human and animal health?. Front. Cell. Infect. Microbiol..

[96] Focà A., Liberto M.C., Quirino A., Marascio N., Zicca E., Pavia G. (2015). Gut inflammation and immunity: What is the role of the human gut virome?. Mediat. Inflamm..

[97] Guo R., Li S., Zhang Y., Zhang Y., Wang G., Ma Y., Yan Q. (2021). Dysbiotic oral and gut viromes in untreated and treated rheumatoid arthritis patients. bioRxiv.

[98] Guan S.M., Fu S.M., He J.J., Zhang M. (2011). *Prevotella intermedia* induces prostaglandin E2 via multiple signaling pathways. J. Dent. Res..

[99] Dubois R.N., Abramson S.B., Crofford L., Gupta R.A., Simon L.S., Van De Putte L.B., Lipsky P.E. (1998). Cyclooxygenase in biology and disease. FASEB J..

[100] Kang R.Y., Freire-Moar J., Sigal E., Chu C.Q. (1996). Expression of cyclooxygenase-2 in human and an animal model of rheumatoid arthritis. Br. J. Rheumatol..

[101] Tanaka S., Yoshida M., Murakami Y., Ogiwara T., Shoji M., Kobayashi S., Watanabe S., Machino M., Fujisawa S. (2008). The relationship of *Prevotella intermedia*, *Prevotella nigrescens* and *Prevotella melaninogenica* in the supragingival plaque of children, caries and oral malodor. J. Clin. Pediatr. Dent..

[102] Ceccarelli F., Saccucci M., Di Carlo G., Lucchetti R., Pilloni A., Pranno N., Luzzi V., Valesini G., Polimeni A. (2019). Periodontitis and Rheumatoid Arthritis: The Same Inflammatory Mediators?. Mediat. Inflamm..

[103] Ceccarelli F., Orrù G., Pilloni A., Bartosiewicz I., Perricone C., Martino E., Lucchetti R., Fais S., Vomero M., Olivieri M. (2018). *Porphyromonas gingivalis* in the tongue biofilm is associated with clinical outcome in rheumatoid arthritis patients. Clin. Exp. Immunol..

[104] Arvikar S.L., Collier D.S., Fisher M.C., Unizony S., Cohen G.L., McHugh G., Kawai T., Strle K., Steere A.C. (2013). Clinical correlations with *Porphyromonas gingivalis* antibody responses in patients with early rheumatoid arthritis. Arthritis Res. Ther..

[105] FAO/WHO (2001). Health and nutritional properties of probiotics in food including powder milk with live lactic acid bacteria. Prevention.

[106] Probiotics: What You Need To Know. https://www.nccih.nih.gov/health/probiotics-what-you-need-to-know.

[107] Kato-Kataoka A., Nishida K., Takada M., Kawai M., Kikuchi-Hayakawa H., Suda K., Ishikawa H., Gondo Y., Shimizu K., Matsuki T. (2016). Fermented Milk Containing *Lactobacillus casei* Strain Shirota Preserves the Diversity of the Gut Microbiota and Relieves Abdominal Dysfunction in Healthy Medical Students Exposed to Academic Stress. Appl. Environ. Microbiol..

[108] Sharif A., Kashani H.H., Nasri E., Soleimani Z., Sharif M.R. (2017). The Role of Probiotics in the Treatment of Dysentery: A Randomized Double-Blind Clinical Trial. Probiotics Antimicrob. Proteins.

[109] McFarland L.V., Evans C.T., Goldstein E.J.C. (2018). Strain-Specificity and Disease-Specificity of Probiotic Efficacy: A Systematic Review and Meta-Analysis. Front. Med..

[110] Corcoran B.M., Stanton C., Fitzgerald G.F., Ross R.P. (2005). Survival of probiotic lactobacilli in acidic environments is enhanced in the presence of metabolizable sugars. Appl. Environ. Microbiol..

[111] Amdekar S., Singh V., Singh R., Sharma P., Keshav P., Kumar A. (2011). *Lactobacillus casei* reduces the inflammatory joint damage associated with collagen-induced arthritis (CIA) by reducing the proinflammatory cytokines: *Lactobacillus casei*: COX-2 inhibitor. J. Clin. Immunol..

[112] Pan X., Chen F., Wu T., Tang H., Zhao Z. (2009). The acid, bile tolerance and antimicrobial property of *Lactobacillus acidophilus* NIT. Food Control.

[113] Bernet-Camard M.F., Liévin V., Brassart D., Neeser J.R., Servin A.L., Hudault S. (1997). The human Lactobacillus acidophilus strain LA1 secretes a nonbacteriocin antibacterial substance(s) active in vitro and in vivo. Appl. Environ. Microbiol..

[114] Hooper L.V., Wong M.H., Thelin A., Hansson L., Falk P.G., Gordon J.I. (2001). Molecular analysis of commensal host-microbial relationships in the intestine. Science.

[115] Perdigón G., de Macias M.E., Alvarez S., Oliver G., de Ruiz Holgado A.P. (1988). Systemic augmentation of the immune response in mice by feeding fermented milks with *Lactobacillus casei* and *Lactobacillus acidophilus*. Immunology.

[116] Sun Y., Chen Q., Lin P., Xu R., He D., Ji W., Bian Y., Shen Y., Li Q., Liu C. (2019). Characteristics of gut microbiota in patients with rheumatoid arthritis in Shanghai, China. Front. Cell. Infect. Microbiol..

[117] Chang H.Y., Tang F.Y., Chen D.Y., Chih H.M., Huang S.T., Cheng H.D., Lan J.L., Chiang E.P. (2013). Clinical use of cyclooxygenase inhibitors impairs vitamin B-6 metabolism. Am. J. Clin. Nutr..

[118] Sande J.S., Ulvik A., Midttun Ø., Ueland P.M., Hammer H.B., Valen M., Apalset E.M., Gjesdal C.G. (2019). Vitamin B-6 Status Correlates with Disease Activity in Rheumatoid Arthritis Patients During Treatment with TNFα Inhibitors. J. Nutr..

[119] Woolf K., Manore M.M. (2008). Elevated plasma homocysteine and low vitamin B-6 status in nonsupplementing older women with rheumatoid arthritis. J. Am. Diet. Assoc..

[120] Karczewski J., Troost F.J., Konings I., Dekker J., Kleerebezem M., Brummer R.J., Wells J.M. (2010). Regulation of human epithelial tight junction proteins by *Lactobacillus plantarum* in vivo and protective effects on the epithelial barrier. Am. J. Physiol. Gastrointest. Liver Physiol..

[121] Lam E.K.Y., Tai E.K.K., Koo M.W.L., Wong H.P.S., Wu W.K.K., Yu L., So W.H.L., Woo P.C.Y., Cho C.H. (2007). Enhancement of gastric mucosal integrity by *Lactobacillus rhamnosus* GG. Life Sci..

[122] Mangell P., Nejdfors P., Wang M., Ahrné S., Weström B., Thorlacius H., Jeppsson B. (2002). *Lactobacillus plantarum* 299v inhibits *Escherichia coli*-induced intestinal permeability. Dig. Dis. Sci..

[123] Eizaguirre I., Urkia N.G., Asensio A.B., Zubillaga I., Zubillaga P., Vidales C., Garcia-Arenzana J.M., Aldazabal P. (2002). Probiotic supplementation reduces the risk of bacterial translocation in experimental short bowel syndrome. J. Pediatr. Surg..

[124] Hong S.W., Kim J.H., Bae H.J., Ham J.S., Yoo J.G., Chung K.S., Oh M.H. (2018). Selection and characterization of broad-spectrum antibacterial substance-producing *Lactobacillus curvatus* PA40 as a potential probiotic for feed additives. Anim. Sci. J..

[125] De Keersmaecker S.C., Verhoeven T.L., Desair J., Marchal K., Vanderleyden J., Nagy I. (2006). Strong antimicrobial activity of *Lactobacillus rhamnosus* GG against *Salmonella typhimurium* is due to accumulation of lactic acid. FEMS Microbiol. Lett..

[126] Markowiak-Kopeć P., Śliżewska K. (2020). The Effect of Probiotics on the Production of Short-Chain Fatty Acids by Human Intestinal Microbiome. Nutrients.

[127] Zamani B., Golkar H.R., Farshbaf S., Emadi-Baygi M., Tajabadi-Ebrahimi M., Jafari P., Akhavan R., Taghizadeh M., Memarzadeh M.R., Asemi Z. (2016). Clinical and metabolic response to probiotic supplementation in patients with rheumatoid arthritis: A randomized, double-blind, placebo-controlled trial. Int. J. Rheum. Dis..

[128] Wang L., Zhang H., Rehman M.U., Mehmood K., Jiang X., Iqbal M., Tong X., Gao X., Li J. (2018). Antibacterial activity of *Lactobacillus plantarum* isolated from Tibetan yaks. Microb. Pathog..

[129] Hitchon C.A., El-Gabalawy H.S. (2004). Oxidation in rheumatoid arthritis. Arthritis Res. Ther..

[130] Kamanli A., Naziroğlu M., Aydilek N., Hacievliyagil C. (2004). Plasma lipid peroxidation and antioxidant levels in patients with rheumatoid arthritis. Cell Biochem. Funct..

[131] Alipour B., Homayouni-Rad A., Vaghef-Mehrabany E., Sharif S.K., Vaghef-Mehrabany L., Asghari-Jafarabadi M., Nakhjavani M.R., Mohtadi-Nia J. (2014). Effects of *Lactobacillus casei* supplementation on disease activity and inflammatory cytokines in rheumatoid arthritis patients: A randomized double-blind clinical trial. Int. J. Rheum. Dis..

[132] Vaghef-Mehrabany E., Alipour B., Homayouni-Rad A., Sharif S.K., Asghari-Jafarabadi M., Zavvari S. (2014). Probiotic supplementation improves inflammatory status in patients with rheumatoid arthritis. Nutrition.

[133] Vaghef-Mehrabany E., Homayouni-Rad A., Alipour B., Sharif S.K., Vaghef-Mehrabany L., Alipour-Ajiry S. (2016). Effects of Probiotic Supplementation on Oxidative Stress Indices in Women with Rheumatoid Arthritis: A Randomized Double-Blind Clinical Trial. J. Am. Coll. Nutr..

[134] Vaghef-Mehrabany E., Vaghef-Mehrabany L., Asghari-Jafarabadi M., Homayouni-Rad A., Issazadeh K., Alipour B. (2017). Effects of probiotic supplementation on lipid profile of women with rheumatoid arthritis: A randomized placebo-controlled clinical trial. Health Promot. Perspect..

[135] Andreasen A.S., Larsen N., Pedersen-Skovsgaard T., Berg R.M., Møller K., Svendsen K.D., Jakobsen M., Pedersen B.K. (2010). Effects of *Lactobacillus acidophilus* NCFM on insulin sensitivity and the systemic inflammatory response in human subjects. Br. J. Nutr..

[136] Zamani B., Farshbaf S., Golkar H.R., Bahmani F., Asemi Z. (2017). Synbiotic supplementation and the effects on clinical and metabolic responses in patients with rheumatoid arthritis: A randomised, double-blind, placebo-controlled trial. Br. J. Nutr..

[137] de los Angeles Pineda M., Thompson S.F., Summers K., de Leon F., Pope J., Reid G. (2011). A randomized, double-blinded, placebo-controlled pilot study of probiotics in active rheumatoid arthritis. Med. Sci. Monit. Int. Med. J. Exp. Clin. Res..

[138] Mandel D.R., Eichas K., Holmes J. (2010). Bacillus coagulans: A viable adjunct therapy for relieving symptoms of rheumatoid arthritis according to a randomized, controlled trial. BMC Complement. Altern. Med..

[139] Hatakka K., Martio J., Korpela M., Herranen M., Poussa T., Laasanen T., Saxelin M., Vapaatalo H., Moilanen E., Korpela R. (2003). Effects of probiotic therapy on the activity and activation of mild rheumatoid arthritis—A pilot study. Scand. J. Rheumatol..

[140] Osorio F., Reis e Sousa C. (2011). Myeloid C-type lectin receptors in pathogen recognition and host defense. Immunity.

[141] Konstantinov S.R., Smidt H., de Vos W.M., Bruijns S.C., Singh S.K., Valence F., Molle D., Lortal S., Altermann E., Klaenhammer T.R. (2008). S layer protein A of *Lactobacillus acidophilus* NCFM regulates immature dendritic cell and T cell functions. Proc. Natl. Acad. Sci. USA.

[142] Lightfoot Y.L., Selle K., Yang T., Goh Y.J., Sahay B., Zadeh M., Owen J.L., Colliou N., Li E., Johannssen T. (2015). SIGNR3-dependent immune regulation by *Lactobacillus acidophilus* surface layer protein A in colitis. EMBO J..

[143] Lebeer S., Bron P.A., Marco M.L., Van Pijkeren J.P., O’Connell Motherway M., Hill C., Pot B., Roos S., Klaenhammer T. (2018). Identification of probiotic effector molecules: Present state and future perspectives. Curr. Opin. Biotechnol..

[144] Duar R.M., Lin X.B., Zheng J., Martino M.E., Grenier T., Pérez-Muñoz M.E., Leulier F., Gänzle M., Walter J. (2017). Lifestyles in transition: Evolution and natural history of the genus *Lactobacillus*. FEMS Microbiol. Rev..

[145] Heeney D.D., Gareau M.G., Marco M.L. (2018). Intestinal *Lactobacillus* in health and disease, a driver or just along for the ride?. Curr. Opin. Biotechnol..

[146] Salminen S., von Wright A., Morelli L., Marteau P., Brassart D., de Vos W.M., Fondén R., Saxelin M., Collins K., Mogensen G. (1998). Demonstration of safety of probiotics—A review. Int. J. Food Microbiol..

[147] Zhang X., Zhang D., Jia H., Feng Q., Wang D., Liang D., Wu X., Li J., Tang L., Li Y. (2015). The oral and gut microbiomes are perturbed in rheumatoid arthritis and partly normalized after treatment. Nat. Med..

[148] Omar A.M., Ahmadi N., Ombada M., Fuscaldo J., Siddiqui N., Safo M., Nalamalapu S. (2019). Breaking Bad: A case of *Lactobacillus* bacteremia and liver abscess. J. Community Hosp. Intern. Med. Perspect..

[149] Salminen M.K., Rautelin H., Tynkkynen S., Poussa T., Saxelin M., Valtonen V., Järvinen A. (2004). *Lactobacillus* bacteremia, clinical significance, and patient outcome, with special focus on probiotic *L. rhamnosus* GG. Clin. Infect. Dis..

[150] Sherid M., Samo S., Sulaiman S., Husein H., Sifuentes H., Sridhar S. (2016). Liver abscess and bacteremia caused by lactobacillus: Role of probiotics? Case report and review of the literature. BMC Gastroenterol..

[151] Tarantino G., Finelli C. (2015). Systematic review on intervention with prebiotics/probiotics in patients with obesity-related non-alcoholic fatty liver disease. Future Microbiol..

[152] Tuomola E., Crittenden R., Playne M., Isolauri E., Salminen S. (2001). Quality assurance criteria for probiotic bacteria. Am. J. Clin. Nutr..

[153] Vanhee L.M., Goemé F., Nelis H.J., Coenye T. (2010). Quality control of fifteen probiotic products containing *Saccharomyces boulardii*. J. Appl. Microbiol..

[154] Cocetta V., Catanzaro D., Borgonetti V., Ragazzi E., Giron M.C., Governa P., Carnevali I., Biagi M., Montopoli M. (2019). A Fixed Combination of Probiotics and Herbal Extracts Attenuates Intestinal Barrier Dysfunction from Inflammatory Stress in an In vitro Model Using Caco-2 Cells. Recent Pat. Food Nutr. Agric..

[155] Riedel C.U., Foata F., Goldstein D.R., Blum S., Eikmanns B.J. (2006). Interaction of bifidobacteria with Caco-2 cells-adhesion and impact on expression profiles. Int. J. Food Microbiol..

